# Regional Variation in Health Care Utilization Among Adults With Inadequate Cardiovascular Health in the USA

**DOI:** 10.7759/cureus.44121

**Published:** 2023-08-25

**Authors:** Abraham M Enyeji, Noel C Barengo, Gilbert Ramirez, Boubakari Ibrahimou, Alejandro Arrieta

**Affiliations:** 1 Department of Global Health, Robert Stempel College of Public Health & Social Works, Florida International University, Miami, USA; 2 Faculty of Medicine, Riga Stradiņš University, Riga, LVA; 3 Department of Translational Medicine, Herbert Wertheim College of Medicine, Miami, USA; 4 Department of Biostatistics, Robert Stempel College of Public Health & Social Works, Florida International University, Miami, USA

**Keywords:** fpl: federal poverty level, nh: non-hispanic, aha: american heart association, cvh: cardiovascular health, cvd: cardiovascular disease

## Abstract

Background

Prior evidence of region-level differences in health outcomes and specialized healthcare services in the US poses questions of whether there are differences in utilization of healthcare that may account for regional differences in healthcare outcomes. This study aimed to examine regional differences in healthcare utilization for individuals with poor cardiovascular health (CVH) compared to those with ideal/intermediate CVH.

Methods

In this cross-sectional analytical study, two 3-year periods (2008-2010 and 2018-2020) were pooled and analyzed using multivariate Poisson’s regression of region on counts of healthcare utilization, while controlling for relevant covariates. The interaction of the non-southern regions with recent years was to reveal how the regional dispersion in healthcare usage was changing over time for the non-southern regions compared to the south.

Results

The results showed significant regional variation in healthcare usage for individuals with poor CVH, with lower health utilization rates observed primarily in southern states, consistent with higher rates of coronary heart disease in those regions. The impact of a unit improvement on CVH score was to reduce the level of healthcare utilization by 15.7% ([95% CI, 15 - 17%; p < 0.001]) for individuals with poor CVH and 19.1% ([95% CI, 19 - 20%; p < 0.001]) for the intermediate and ideal subgroups, with the Northeast exhibiting the highest level of healthcare usage.

Conclusion

Our results suggest that there is a need for public health interventions to reduce regional disparities in access to healthcare for the people at greatest risk of cardiovascular events by considering individual factors as well as the broader regional and policy contexts where these people live.

## Introduction

Despite significant efforts made over decades to improve the mortality rate related to cardiovascular disease (CVD), it continues to be the primary cause of death and morbidity in the United States. In the year 2019, coronary heart disease (CHD) accounted for the highest proportion (43.1%) of CVD-related deaths in the United States. This was followed by other forms of CVD (17.3%), stroke (17.2%), high blood pressure (11.7%), heart failure (9.9%), and disease of the arteries (2.8%) [1]. In response to this trend, the American Heart Association (AHA) has recently formulated a strategic goal to address the issue, aiming for an equitable increase in healthy life expectancy, from 66 to at least 68 years of age across the United States (US) and from 64 to at least 67 years of age worldwide [2]. The concept of cardiovascular health (CVH) was, therefore, introduced by the AHA to assess progress towards this goal. Previous studies have shown that the number of ideal CVH metrics strongly predicts CVD mortality. For example, individuals with ideal CVH scores experienced a significant 78% lower risk of all-cause mortality and a 59% lower risk of cardiovascular disease (CVD) when compared to those with poor CVH scores [3,4].

A recent study conducted by Kolte et al. (2015) identified the Midwest as having the lowest incidence of in-hospital cardiac arrest (IHCA) and the highest survival-to-hospital discharge ratio. However, contrasting findings revealed that the highest rates of in-hospital cardiac arrests were observed in the West [5]. These discrepancies highlight the complexity of IHCA incidence, which is influenced by various factors including the severity of patients' illnesses, institutional response and care processes for acutely and chronically ill patients, as well as patients' willingness and ability to seek medical care.

 It is well-established that individuals with poor CVH scores are at a higher risk of CVD-related mortality. Therefore, it becomes crucial to examine whether differences in overall healthcare utilization across the four U.S. census regions can explain the variations in health outcomes [6]. By investigating healthcare utilization patterns, we can gain insights into potential contributing factors to regional health disparities and develop targeted interventions to improve outcomes for individuals at risk of CVD.

Concerns have arisen regarding regional disparities in healthcare usage and their potential impact on health outcomes, raising questions about the efficiency of healthcare supply [7]. However, a comprehensive examination of regional disparities in healthcare utilization for individuals with poor cardiovascular health (CVH) at the national level in the United States of America (USA) remains lacking.

This study aims to fill this gap by assessing the regional variation in healthcare utilization in the USA, comparing data from previous years (2008-2010) with more recent years (2017, 2018, & 2020) specifically for individuals with inadequate or poor CVH status. The goal is to demonstrate the association between the region of residence for patients with poor CVH scores and their level and quality of healthcare utilization. This research is crucial for measuring and interpreting healthcare disparities, particularly between those in greatest need of healthcare services and those who may require them to a lesser extent.

The objective of this study was to understand how regional variations in healthcare utilization impact patients with poor CVH scores and can inform policy and decision-making processes, ultimately leading to more equitable access to healthcare services and improved health outcomes for those most in need.

## Materials and methods

Study design

This study was an analytical cross-sectional study using secondary data from 2008 to 2020 from the Medical Expenditure Panel Survey (MEPS) database. The MEPS comprises extensive nationwide surveys involving households, individuals, insurance companies, health providers (doctors, pharmacies, and hospitals), and employers throughout the United States. It is sponsored by the Agency for Healthcare Research and Quality (AHRQ). The panel is made up of randomly selected non-institutionalized US civilians, providing nationally representative estimates of sociodemographic characteristics, medical conditions, utilization, and healthcare costs [6].

Study population

The final sample consisted of adults aged 40 and above who had a poor cardiovascular health (CVH) management score and also participated in the Medical Expenditure Panel Survey (MEPS) between 2008 and 2020. To create this sample, two MEPS components were merged: (1) the household component full-year files, which included demographic characteristics; and (2) the medical conditions files which provided a “current” health status of each individual at any point during the data year. The household component events file included provider visits related to any specific health condition. The data files were merged using person identifiers for each year. Combining the merged data from two three-year periods, encompassing the years 2008-2010 and 2018-2020, allowed for a larger sample of adults with poor CVH. The event types analyzed in MEPS included inpatient, outpatient, office-based, and emergency room visits. Events related to dental visits were excluded from this study.

Primary outcome

The main outcome variable was the total number of healthcare service utilization events per person per year. This variable was generated from multiple service categories that included inpatient, outpatient, office-based, and emergency room visits. MEPS collects this information from medical providers, insurance companies, employers, and hospitals. 

Primary independent variable

The primary independent variables of interest in this study were the regional variables: Northeast, West, Midwest, and South. These geographical region variables, based on the four census regions in the United States, were examined to assess their potential impact on regional differences in healthcare utilization among individuals with poor cardiovascular health (CVH).

Non-dietary cardiovascular health 

The CVH composite score, ranging from 0 to 6, represents the number of achieved CVH scores for each participant. This study examined non-dietary CVH components, including inadequate physical activity, obesity, smoking, hypercholesterolemia, hypertension, and diabetes mellitus. Diet was not assessed in the MEPS data. Participants' CVH status was determined using responses from a self-administered questionnaire, classifying individuals with a binary variable as either "favorable" (assigned a value of 1) or "unfavorable" (assigned a value of 0). Therefore, the maximal score of 6 was achieved as a composite score, as did other studies [8]. Those with diagnosed cholesterol disorder, hypertension, diabetes mellitus, recent smoking, insufficient physical activity, or BMI >25 or <18 kg/m2 were classified as unfavorable risk factors and assigned a value of 0. Participants without these adverse pre-conditions were assigned a value of 1. The CVH score for each participant was then obtained by summing the assigned values, resulting in scores ranging from 0 to 6.

Control variables

Age, education level, insurance status, sex, race/ethnicity, and family income were examined as factors associated with health expenditures. Therefore, they were investigated as covariates to determine the relationship between CVH scores and the average number of hospital events, for individuals with poor vs. those with intermediate to ideal CVH profiles. Participants' sex was categorized as self-reported male or self-reported female. Self‐reported races or ethnicities were classified as White (non‐Hispanic), Black (non‐Hispanic), Hispanic, and Asian (non‐Hispanic). Family income levels were defined as follows: poor or very low income (<125% of the federal poverty level), low income (125% to < 200% of the federal poverty level), middle income (200% to < 400% of the federal poverty level), and high income (≥400% of the federal poverty level). Educational levels were categorized as having less than a bachelor’s degree, having achieved the equivalent of a bachelor’s or master’s degree, and ultimately having a doctoral or professional degree. Health insurance status was divided into private, public (Medicare and Medicaid), and uninsured.

Statistical analysis

Descriptive statistics were utilized to pool the six years of MEPS data from 2008-2010 and 2018-2020. The data was summarized to present frequency distributions along with corresponding weighted proportions. Demographic files were obtained for each year and health utilization counts were generated from a combination of health events datasets that included inpatient, outpatient, office-based, and emergency room visits for each year. The demographics file was merged with health event files using key variables and the data was pooled for all six years. First, a Poisson’s regression was run of healthcare event counts on regionality, specifying high-risk populations while controlling for age, sex, race/ethnicity, insurance status, level of income, and education.

The interaction of the non-South with recent years was to be flexible about the impact of how health visits changed for the non-South relative to the southern region (reference group).

## Results

Characteristics of the sample

The sample used consisted of 120,044 MEPS participants across the years 2008-2020 who were more than 40 years of age and either belonged to the high-risk or intermediate/low-risk group. Individuals considered in high-risk groups had poor CVH scores, while intermediate and low-risk groups had intermediate and ideal CVH scores. The distribution of means of proportions in demographic characteristics for the study population as a function of the covariates is shown in Table 1 below:

**Table 1:** Summary statistics of study participants across the years 2008-2020 who were 40 years or older. Means and proportions were compared across all groups. Confidence Interval in parenthesis. Abbreviations - FPL: Federal poverty level; MWest: Mid West; NEast: Northeast; Private: Private insurance.

Proportions % (95% CI)	High Risk Gr. % (95% CI)	Intermediate/ Low Risk Gr.	Diff (p-values)	Combined Sample % (95% CI)
Whites	0.712 (0.71 - 0.72)	0.76 (0.75 - 0.76)	P<0.001	0.75 (0.75 - 0.75)
Blacks	0.22 (0.22 - 0.23)	0.16 (0.16 - 0.17)	P<0.001	0.18 (0.18 - 0.18)
Asians	0.03 (0.03 - 0.04)	0.06 (0.06 - 0.06)	P<0.001	0.05 (0.05 - 0.05)
Hispanics	0.17 (0.16 - 0.18)	0.21 (0.21 - 0.22)	P<0.001	0.20 (0.19 - 0.21)
Male	0.46 (0.45 - 0.47)	0.46 (0.46 - 0.46)	P<0.001	0.460 (0.457 - 0.463)
US Region				
West	0.20 (0.19 - 0.21)	0.26 (0.26 - 0.27)	P<0.001	0.25 (0.25 - 0.25)
MWest	0.21 (0.20 - 0.21)	0.20 (0.20 - 0.21)	P<0.001	0.20 (0.20 - 0.21)
NEast	0.157 (0.15 - 0.16)	0.16 (0.15 - 0.16)	P<0.001	0.16 (0.15 - 0.16)
Insurance status				
Private	0.50 (0.49 - 0.51)	0.65 (0.64 - 0.66)	P<0.001	0.61 (0.61 - 0.62)
Medicare/Medicaid	0.44 (0.43 - 0.45)	0.24 (0.23 - 0.24)	P<0.001	0.29 (0.28 - 0.29)
Uninsured	0.06 (0.05 - 0.07)	0.11 (0.11 - 0.11)	P<0.001	0.09 (0.09 - 0.10)
Age Groups				
Age 40-64	0.15 (0.15 - 0.16)	0.48 (0.48 – 0.49)	P<0.001	0.332 (0.33 - 0.33)
Age 65-79	0.34 (0.33 - 0.35)	0.18 (0.18 - 0.18)	P<0.001	0.22 (0.21 - 0.22)
Age > 80	0.11 (0.10 – 0.11)	0.06 (0.05 – 0.06)	P<0.001	0.07 (0.7 - 0.07)
Family Income category (Federal Poverty Level)				
<125% FPL	0.07 (.07 - 0.08)	0.11 (0.11 - 0.12)	P<0.001	0.09 (0.10 - 0.10)
125-200% FPL	0.05 (0.04 - 0.06)	0.09 (0.09 - 0.09)	P<0.001	0.07 (0.07 - 0.07)
200-400% FPL	0.0 (0.08 - 0.09)	0.197 (0.196 - 0.201)	P<0.001	0.15 (0.14 - 0.15)
>400% FPL	0.15 (0.15 - 0.16)	0.60 (0.59 - 0.60)	P<0.001	0.40 (0.39 - 0.40)
Highest Educational level attained				
Bachelor's/Master’s degree	0.17 (0.16 - 0.18)	0.26 (0.26 - 0.27)	P<0.001	0.24 (0.24 - 0.24)
PhD or Professional Degree	0.03 (0.02 - 0.03)	0.05 (0.04 - 0.05)	P<0.001	0.04 (0.04 - 0.04)
Sample size	55,218	64,826	P<0.001	120,044

In the above table, Asians, Whites, and Hispanics are relatively under-represented in the high-risk sub-sample, while Blacks are overrepresented. By contrast, the percentage of participants self-identifying as male is virtually the same in both sub-samples at 46%. For regions, the Northeast is underrepresented in the high-risk sub-sample, while the South is overrepresented. Those with public insurance are over-represented in the high-risk sub-sample, while those with private insurance or no insurance are underrepresented in the high-risk sample. Unsurprisingly, those aged between 65 and 79 years or 80 or higher are over-represented in the high-risk sub-sample. The elderly and those with incomes below or at 125% of the Federal poverty level are at higher risk of being in the high-risk sub-group. All the above poverty-level income groups are relatively underrepresented in the high-risk sub-group, in addition to more educated people.

In the first pair of regressions in Table 2, on average, across all six years, for the high-risk individuals, the impact of a unit improvement on CVH score was to reduce the level of healthcare utilization by 15.7% for both the regressions (p-value < 0.001). Similarly, a unit improvement in CVH scores is associated with an even higher drop in healthcare utilization (19.1%) for those at intermediate or low risk. This suggests that improvements in CVH scores are robustly associated with reducing the frequency of health visits of any kind for both groups, but more so those with intermediate to ideal CVH scores. Similarly, the regional variations of differences in healthcare utilization were qualitatively the same but more pronounced for intermediate or low-risk individuals versus the high-risk group. The regional differences in medical visits for the high-risk groups include; those in the Northeast used services at an average of 16.9% higher than individuals with similar characteristics from the South, the reference group. Westerners followed closely with a 13.2% higher healthcare usage, and the Midwest provided a 10.2% higher health usage than the South. These results tie in with findings from previous researchers. As an illustration, the southern states exhibited higher rates of poor CVH and mortality due to cardiovascular disease. Additionally, poor CVH was strongly associated with minority groups and low socioeconomic status. [9]. Furthermore, states with poor or intermediate CVH scores were mostly clustered in the southern United States [10].

**Table 2:** Results for the predicted annual number of healthcare visits by CVH scores based on Region for the years 2008-2020 using the MEPS dataset. Abbreviations - CVH: Cardiovascular Health; FPL: Federal poverty level; Education BA/MA: Education bachelor’s and master’s degrees; MEPS: Medical Expenditure Panel Survey; (*) Represents the interaction term.

Sample	CVH <=2 (95% CI)				CVH => 2 (95% CI)		
	Adjusted Spec.	Interaction effects	P-Value	Adjusted Spec.		Interaction effects	P-Value
Constant	1.947 (1.91 - 1.98)	1.935 (1.90 - 1.97)	P<0.001	1.658 (1.63 - 1.68)		1.649 (1.62 - 1.67)	P<0.001
CVH metrics	-0.157 (-0.17 - 0.15)	-0.157 (-0.17 - -0.15)	P<0.001	-0.191 (-0.20 - -0.19)		-.191 (-0.19 -0.20)	P<0.001
Northeast	0.169 (0.16 - 0.18)	0.130 (0.11 - 0.15)	P<0.001	0.212 (0.20 - 0.22)		0.235 (0.22 - 0.25)	P<0.001
Northeast * Trend	-	0.005 (0.00 - 0.01)		-		-0.003 (-.005 - -.001)	P<0.01
Midwest	0.102 (0.10 - .11)	0.153 (0.13 - 0.17)	P<0.001	0.158 (0.15 - 0.17)		0.187 (0.17 - 0.21)	P<0.001
Midwest * Trend	-	-.006 (-0.01 - 0.00)		-		-0.003 (-.006 - -.002)	P<0.001
West	0.132 (0.12 - 0.14)	0.167 (0.15 - 0.19)	P<0.001	0.172 (0.16 - 0.18)		0.169 (0.15 - 0.19)	P<0.001
West * Trend	-	-.004 (-0.01 - 0.00)	P<0.001	-		.0002 (-0.002 - 0.002)	0.762
Family Income: 125-200% FPL	-0.111 (-0.12 - -0.10)	-0.111 (-0.12 - -0.10)	P<0.001	-0.055 (-0.07 - -0.04)		-0.054 (-0.06 - -0.04)	P<0.001
Family Income: 200-400% FPL	-.147 (-0.16 - -0.14)	-.147 (-0.16 - 0.14)	P<0.001	-0.054 (-0.07 - -0.04)		-0.055 (-0.06 - -0.04)	P<0.001
Family Income: 400% + FPL	-0.082 (-0.10 - -0.07)	-0.082 (-0.10 - -0.07)	P<0.001	0.044 (0.03 - 0.06)		0.044 (0.03 - 0.06)	P<0.001
Ages 65-79	0.108 (0.10 - 0.12)	0.108 (-0.10 - -0.07)	P<0.001	0.304 (0.29 - 0.31)		0.304 (0.29 - 0.31)	P<0.001
Ages 80+	0.050 (0.03 - 0.06)	0.049 (0.04 - 0.06)	P<0.001	0.324 (0.31 - 0.33)		0.324 (0.31 - 0.34)	P<0.001
Male	-0.128 (-0.14 - -0.12)	-0.129 (-0.14 - -0.13)	P<0.001	-0.292 (-0.29 - -0.28)		-0.292 (-0.30 - -0.29)	P<0.001
Hispanic	-0.293 (-0.31 - -0.28)	-0.292 (-0.31 - -0.28)	P<0.001	-0.475 (-0.48 - -0.46)		-0.475 (-0.49 - -0.46)	P<0.001
Black	-0.136 (-0.15 - -0.12)	-0.135 (-0.15 - -0.12)	P<0.001	-0.292 (-0.30 - -0.28)		-0.292 (-0.30 - -0.28)	P<0.001
Asian	-0.499 (-0.52 - 0.50)	-0.499 (-0.53 - -0.47)	P<0.001	-0.563 (-0.58 - -0.54)		-0.562 (-0.58 - -0.54)	P<0.001
Education BA/MA	0.190 (0.18 - 0.20)	0.192 (0.18 - 0.20)	P<0.001	0.181 (0.17 - 0.19)		0.181 (0.17 - 0.19)	P<0.001
Education PhD/Completed	0.276 (0.24 - 0.31)	0.276 (0.24 - 0.30)	P<0.001	0.212 (0.19 - 0.23)		0.212 (0.19 - 0.23)	P<0.001
Trend	0.019 (0.02 - 0.02)	0.021 (0.02 - 0.02)	P<0.001	0.026 (0.02 - 0.03)		0.027 (0.02 - 0.03)	P<0.001
Medicaid/Medicare	0.930 (0.90 - 0.96)	0.932 (0.90 - 0.96)	P<0.001	1.10 (1.08 - 1.12)		1.10 (1.08 - 1.12)	P<0.001
Private Insurance	0.748 (0.72 - 0.78)	0.750 (0.72 - 0.78)	P<0.001	0.933 (0.91 - 0.95)		0.932 (0.91 - 0.95)	P<0.001
Pseudo R-squared (Sample size)	0.0651 (15,919)	0.0654 (15,919)	P<0.001	0.1376 (43,973)		0.1310 (43,973)	P<0.001

F-Tests were conducted to test for differences between the means of coefficients for regions because their coefficients were similar but distinct. The null hypothesis of no differences across the non-South regions in the mean visits was rejected, with the joint F-Test that the three region dummies all having the same value yielding a p-value of less than .05.

The other control variables also yielded intriguing results. The three relative income controls were derived from dividing reported income by the Federal poverty level (FPL). Individuals with higher incomes consistently showed lower utilization of healthcare services compared to those who were at or near the Federal poverty level.

Individuals with an income level exceeding 2.5 times the Federal poverty level exhibited a 14.7% lower rate of health facility utilization compared to those at the poverty level. Similarly, individuals with income well above the poverty level tended to experience similarly low rates of healthcare utilization, i.e., they had an 8.2% lower use of medical services than those at the FPL (p-values < 0.001). The high-risk population groups associated with public insurance (Medicaid & Medicare) showed a greater frequency of utilization of health services on average, percentage-wise, compared to privately insured participants regarding how far their scores were above the mean scores of the uninsured high-risk groups. This suggests that the uninsured individuals with poor CVH scores were likely to have half as many visits to a healthcare provider than their counterparts who were publicly or privately insured.

As expected, individuals with a college education or higher had a higher frequency of healthcare visits compared to those who attained an associate degree or lower. These results substantiate the findings of previous researchers. Some suggested a substantial improvement in the use of maternal health services could be reached by accelerating socioeconomic development and promoting the needs of schooling, and economic welfare [11]. Participants from all regions other than the South, however, demonstrated a higher tendency to utilize medical care; i.e., engaging to a greater extent in healthcare treatment, particularly those in the northeastern region showed the most frequency of visits for the high-risk groups (p-value<0.001). The p-values of these estimates were significant at the 1% level of significance.

The impact of the interaction of a trend variable with region dummy variables was to reveal how the regional dispersion in healthcare usage was changing over time. The trends together show that average percentage changes in the frequency of health-seeking behavior tend to be slowly converging across the regions by virtue of it rising at a modestly faster rate in the South than in the other regions. The biggest exception to this is how in the Northeast, among the high-risk sub-group, the percentage increase was 0.5 higher than in the South. The percentage increase over time is also higher for the less risky sub-group which may reflect the fact that they needed fewer healthcare visits initially. This would explain why the coefficients for the second pair of regressions tend to be consistently bigger in magnitude than the first pair of regressions in Table 2. So, the interaction effects of the trend with the West and Midwest showed a yearly convergence of health-seeking behavior by 0.4% or 0.6%, respectively, among high-risk groups, while those in the Northeast are accelerating healthcare usage by their high-risk individuals by 0.5%/year. By contrast, for the intermediate or low-risk sub-group, the yearly convergence in health-seeking behavior for the Northeast and Midwest was 0.3%, with no sign of convergence between individuals in the West and the South, with an insignificant .02% increase for the West.

**Figure 1:**
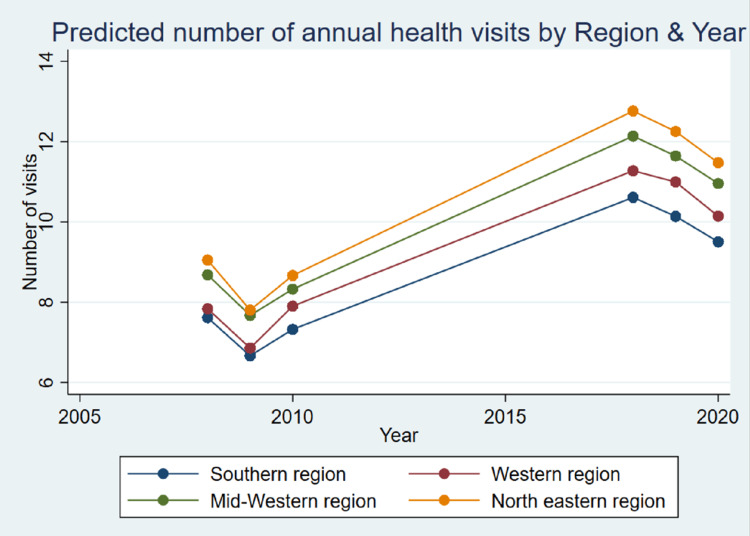
The predicted number of annual healthcare visits by designated region.

Figure 1 shows that the Northeastern region had the highest number of healthcare visits across previous and recent years. Meanwhile, the southern states consistently exhibited the lowest level of health-seeking behavior across all years. It was noted that there was an initial drop in annual average health visits from 2008 to 2009 (from nine to about eight visits/year for the Northeast), followed by a subsequent rise to somewhat similar values in 2010. In more recent years, 2018 showed the highest number of visits for all regions, with a steady decline in average health-seeking behavior for all US census regions approaching more recent years.

## Discussion

As far as our knowledge goes, this study is the first to examine regional disparities in health-seeking behavior among individuals with poor CVH, between 2008 and 2020, using a nationally representative sample. Our findings reveal substantial variation in the prevalence of poor CVH across the four census regions, with a 10-17% higher prevalence in non-southern states compared to the South. These findings align with higher rates of CHD observed in those regions. Furthermore, the prevalence of healthcare utilization demonstrated similar patterns, with the highest rates of healthcare visits in the Northeast and the lowest rates clustered among the southern states, consistent with previous research [9].

Additionally, we observed that socioeconomic indicators, such as the highest level of education attained, were positively associated with a higher frequency of healthcare visits for both high- and low-risk individuals across all regions. Similarly, individuals with poor CVH living in the South demonstrated the lowest frequencies of healthcare visits, even after controlling for other characteristics, which is in line with previous findings [9, 12] that highlighted higher rates of poor CVH and cardiovascular disease mortality in the southern states. In comparing in-hospital mortality rates among individuals with acute myocardial infarctions, they found lower rates in the Midwest and West regions, but higher rates in the South [13].

The regional differences in healthcare use remained persistent across the four regions, albeit with some convergence over time, as high-risk individuals in the South experienced a slightly higher growth rate compared to low-risk individuals in the Midwest and West. The main exception to this trend was observed in the northeastern region, where individuals with poor CVH showed a higher rate of increase than their southern counterparts, despite starting with the highest level of access among the four regions. This may be attributed to the impact of the Affordable Care Act, which was implemented more consistently in the northeastern region during this time period.

Limitations

It is important to note that these analyses are based on observational, cross-sectional data, which only allow for the examination of associations and not causal relationships between regionality and frequency of healthcare use. Additionally, due to limitations of the MEPS dataset, region-level detail on the frequency distributions of health visits was limited, and the variables for region were restricted to the four US census regions rather than individual states.

## Conclusions

Our findings highlight notable disparities in healthcare utilization among individuals with poor CVH scores at the regional level. The lowest rates of medical care usage were observed in southern states, which aligns with higher rates of coronary heart disease and stroke mortality in those regions. These results emphasize the urgent need for national public health interventions aimed at reducing regional disparities in healthcare access for individuals at the highest risk of cardiovascular events. To effectively address this issue, interventions should consider both individual factors and the broader regional and policy contexts in which these individuals reside. By adopting a comprehensive approach, we can work towards ensuring equitable access to healthcare for all, particularly those most vulnerable to cardiovascular diseases.
